# Primer sets for cloning the human repertoire of T cell Receptor Variable regions

**DOI:** 10.1186/1471-2172-9-50

**Published:** 2008-08-29

**Authors:** Ilenia Boria, Diego Cotella, Irma Dianzani, Claudio Santoro, Daniele Sblattero

**Affiliations:** 1 Department of Medical Sciences and Interdisciplinary Research Centre for Autoimmune Diseases (IRCAD), via Solaroli 17, 28100, Novara, Italy

## Abstract

**Background:**

Amplification and cloning of naïve T cell Receptor (TR) repertoires or antigen-specific TR is crucial to shape immune response and to develop immuno-based therapies. TR variable (V) regions are encoded by several genes that recombine during T cell development. The cloning of expressed genes as large diverse libraries from natural sources relies upon the availability of primers able to amplify as many V genes as possible.

**Results:**

Here, we present a list of primers computationally designed on all functional TR V and J genes listed in the IMGT^®^, the ImMunoGeneTics information system^®^. The list consists of unambiguous or degenerate primers suitable to theoretically amplify and clone the entire TR repertoire. We show that it is possible to selectively amplify and clone expressed TR V genes in one single RT-PCR step and from as little as 1000 cells.

**Conclusion:**

This new primer set will facilitate the creation of more diverse TR libraries than has been possible using currently available primer sets.

## Background

The T cell receptor (TR) is a complex of trans-membrane dimeric proteins that mediate the antigen-dependent activation of T cells [1]. TR recognize self-MHC molecules presenting 'foreign-looking' protein fragments on the surface of infected, cancerous or 'non-self' cells. Most of circulating T cells express TR comprising of alpha and beta chains, while a minimal portion express the gamma and delta dimers [2]. Each chain consists in its extracellular region of a variable (V) and a constant (C) domain. Like immunoglobulin (IG), TR are encoded by several genes that undergo somatic recombination during T cell development [3]. According to the sequences deposited in IMGT^®^, the ImMunoGeneTics information system^®^, , [4-6], the human TRA locus has 47 TRAV, 50 TRAJ and 1 TRAC genes, whereas the TRB locus has 54 TRBV, 2 TRBD, 14 TRBJ and 2 TRBC genes; the TRD locus has 3 TRDV, 3 TRDD, 4 TRDJ and 1 TRDC genes, whereas the TRG locus has 9 TRGV, 5 TRGJ and 2 TRGC genes.

The hypervariable regions, known as complementarity determining regions (CDR), define antigen-binding specificities the CDR1 and CDR2 being encoded by the V genes whereas the CDR3 result from V-(D)-J recombinations. The combinatorial rearrangement of the V, (D) and J genes and the mechanisms of trimming and N addition accounts for the huge diversity of naïve TR and T cell repertoires.

Defining the TR gene usage in antigen-activated T cells is crucial for shaping the immune response in several physiological and pathological conditions such as inflammation and infectious diseases. Furthermore, the cloning of antigen-specific TR is emerging as a powerful strategy for immune-based therapies in autoimmunity, cancer and vaccination [7,8]. However, cloning and expression of specific TR is still a difficult task. TR has an intrinsic low affinity for its antigen and, as membrane-bound protein, is poorly stable when expressed as recombinant soluble protein. Working on the variable portion of few well defined TR, several authors have reported methods to overcome these problems [9]. Soluble and stable TR have been expressed as single-chains [10], or fused to a coiled coil heterodimerization motif [11] or introducing non native disulphide bond [12]. The affinity of specific TR molecules to their antigens has been improved to picomolar levels either by phage [13] or Yeast [14] display methods.

Different methods have been proposed to investigate TR repertoire including length analysis of TR complementarity-determining region 3 (CDR3), flow cytometry, and immuno-histochemistry [15].

The availability of the IMGT/GENE-DB database [5] comprising all germline genes has fuelled the development of several PCR-based methods for cloning TR repertoires. However, the cloning and analysis of TR is rendered difficult by the diversity of the 5' V gene sequences and by the repertoire complexity. Several authors have reported sets of primers that allow PCR-mediated amplification of V regions [16-19]. However, these primers have been designed to amplify subsets of TR genes or have been used in the analysis of clonal T cell populations [20].

Here we report a novel set of primers predicted to amplify nearly 100% of all functional TR V genes. We show that these primers can amplify transcribed TR V genes from as little as 1000 peripheral blood T cells, allowing a reliable and efficient method to clone TR repertoires.

## Results

### Data analysis and primers design

The creation of large diverse libraries representing the specificities of TR repertoires relies on primers which are able to amplify all sequences coding for functional variable regions. With this aim, we developed a strategy to design a new set of primers that greatly reduces the number of reactions needed to amplify all functional V sequences.

Germline V, D and J gene sequences encoding TRA, TRB, TRD and TRG chains [5,6], were retrieved from the IMGT^® ^information system . Two algorithms, "*TCRAlignment*" and "*TCROligo*" (see M&M), have been developed to analyze 47 TRAV, 54 TRBV, 9 TRGV, 3 TRDV, 50 TRAJ, 14 TRBJ, 5 TRGJ and 4 TRDJ genes. In the first step sequences belonging to each data set were grouped into "families" by the *TCRAlignment *algorithm. The algorithm performs an alignment limited to the first 23 bases of FR1 at the 5' end of each V region sequence (starting at base number 1) or in the last 23 bases, at the 3' end in the case of J genes and group them on the basis of similarities. Sequences are grouped if they share less than two mismatches within the 3' 16 bases. This criteria is applied to either 23, 22, 21, 20 or 19 bases long sequences. In the second step the *TCROligo *algorithm uses these sequence families to design unique or degenerated primers (see M&M) for both the V or J region. With these tools we generated a novel set of primers (Table 1 and 2) that makes theoretically feasible the amplification and cloning of the entire TR repertoire. The variable regions of all functional TRA and TRB chains can be *in silico *amplified by 25 and 17 reactions, respectively, while 4 primer pairs are needed to amplify the 9 TRGV genes (Table 1). We also obtained a reduced set of primers for the poor similar J genes (Table 2), being 39 primer pairs sufficient to amplify 50 TRAJ genes and 9 primer pairs for 14 TRBJ genes.

**Table 1:** TR V Region Forward primers

**OLIGO NAME**	**OLIGO SEQUENCE**	**TRA V GENES OPTIMALLY RECOGNIZED**
	**ALPHA**	

**TRAV1for**	GGA CAA ARC MTT GAS CAG CC	V1-1,V1-2
**TRAV2for**	AAG GAC CAA GTG TTT CAG CC	V2
**TRAV3for**	GCT CAG TCA GTG RCY CAG CC	V3, V8-3
**TRAV4for**	GAT GCT AAG ACC ACM CAG CC	V4, V26-1, V26-2
**TRAV5for**	AGA AAA SAW STG GAG CAG AGT C	V5, V10, V22, V34, V41
**TRAV6for**	AGC CAA AAG ATA GAA CAG AA	V6
**TRAV7for**	GAA AAC CAG GTG GAG CAC AG	V7
**TRAV8for**	GCC CAG TCK GTG ASC CAG CW	V8-1, V8-2, V8-4, V8-6, V8-7
**TRAV9for**	GGA AAT TCA GTG RYC CAG AY	V9-1, V9-2
**TRAV12for**	CAG AAG GAG GTG GAG CAG RAT YC	V12-1, V12-2, V12-3
**TRAV13for**	GGA GAG ART GTG GRG CWG CA	V13-1, V13-2
**TRAV14for**	GCC CAG AAG RTW ACT CAA RC	V14/DV4, V19
**TRAV16for**	GCC CAG ASA GTS ACT CAG YC	V16, V38-1, V38-2/DV8
**TRAV17for**	AGT CAA CAG GGA GAA GAG GA	V17
**TRAV18for**	GGA GAC TCG GTT ACC CAG AC	V18
**TRAV20for**	AAA CAG GAG GTG ACG CAG AKT CC	V20, V21
**TRAV23for**	GGC CAA CAG AAG GAG AAA AG	V23/DV6
**TRAV24for**	GAG CTG AAM GTG GAA CAA AR	V24, V39
**TRAV25for**	GGA CAA CAG GTA ATG CAA AT	V25
**TRAV27for**	ACC CAG CTG CTG GAG CAG AG	V27
**TRAV29for**	AGT CAA CAG AAG AAT GAT GA	V29/DV5
**TRAV30for**	CAA CAA CCA GTG CAG AGT CC	V30
**TRAV35for**	GGT CAA CAG CTG AAT CAG AG	V35
**TRAV36for**	GAA GAC AAG GTG GTA CAA AG	V36/DV7
**TRAV40for**	AGC AAT TCA GTC AAG CAG AC	V40

	**BETA**	**TRBV GENES OPTIMALLY RECOGNIZED**

**TRBV2for**	GAT GCT GAA GTC RCM CAG ACT CC	V2, V16, V23-1
**TRBV3for**	GAT GCW GMT GTT WCC CAG AC	V3-1, V24-1
**TRBV4for**	GAC ACT GRA GTY ACS CAG ACA CC	V4-1, V4-2, V4-3, V12-5
**TRBV5for**	GAG GCT GGA GTC ACH CAA AS	V5-1, V5-3, V9, V5-4, V5-5, V5-6, V5-7, V5-8
**TRBV6for**	GAG CCT GGW GTC ASY CAG AC	V6-1, V6-2, V6-3, V6-5, V6-6, V6-7, V6-8, V6-9,V17
**TRBV7for**	GGT GCT GGA GTY KCC CAG W	V7-1, V7-2, V7-3, V11-2, V7-4, V7-6, V7-7, V7-8, V7-9
**TRBV10for**	GAT GCT GRR ATC ACC CAG R	V6-4, V10-1, V10-2, V10-3
**TRBV11for**	GAA GCT GAA GTT GCC CAG TC	V11-1
**TRBV13for**	GAT GCT GGA GTY ATC CAG TC	V13, V12-3, V12-4
**TRBV14for**	GAA GCT GGA GTK RYT CAG T	V11-3, V14
**TRBV15for**	GAT GCC ATG GTC ATC CAG AA	V15
**TRBV18for**	AAT GCC GGC GTC ATG CAG AA	V18
**TRBV19for**	GAT GGT GGA ATC ACT CAG TC	V19
**TRBV20for**	AGT GCT GTC RTC TCT CAA MA	V20-1, V29-1
**TRBV25for**	GAA GCT GAC ATC TAC CAG AC	V25-1
**TRBV27for**	GAT GTG AAA GTR ACC CAG ARC YC	V27, V28
**TRBV30for**	ACA CTC CAG GCA CAG AGA TA	V30

	**GAMMA**	**TRGV GENES OPTIMALLY RECOGNIZED**

**TRGV1for**	TCT TCC AAC TTG GAA GGG RG	V1, V2, V3, V4, V5, V8
**TRGV9for**	GCA GGT CAC CTA GAG CAA CC	V9
**TRGV10for**	TTA TCA AAA GTG GAG CAG TT	V10
**TRGV11for**	CTT GGG CAG TTG GAA CAA CC	V11

	**DELTA**	**TRDV GENES OPTIMALLY RECOGNIZED**

**TRDV1for**	GCC CAG AAG GTT ACT CAA GC	V1
**TRDV2for**	GCC ATT GAG TTG GTG CCT GA	V2
**TRDV3for**	TGT GAC AAA GTA ACC CAG AG	V3

List of optimal primer sequence as designed with the *TCRAlignment *and *TCROligo *algorithms for the TRAV, TRBV, TRGV and TRDV genes.

**Table 2:** TR J gene reverse primers

**OLIGO NAME**	**OLIGO SEQUENCE**	**J GENES OPTIMALLY RECOGNIZED**
	**ALPHA**	

**TRAJ6rev**	CGG ATG AAC AAT AAG GCT GGT TC	J6
**TRAJ10rev**	GAG TTC CAC TTT TAG CTG AG	J10
**TRAJ11rev**	TGG AGA GAC TAG AAG CAT AG	J11
**TRAJ12rev**	TGG ACT GAC CAG MAG TCK GG	J12, J8
**TRAJ13rev**	TGG GAT GAC TTG GAG CTT TG	J13
**TRAJ15rev**	GGA ACT CAC TGA TAG GTG GG	J15
**TRAJ16rev**	AAG ATC CAC CTT TAA CAT GG	J16
**TRAJ17rev**	TGG TTT AAC TAG CAC CCT GG	J17
**TRAJ20rev**	TGC TCT TAC AGT TAC TGT GG	J20
**TRAJ21rev**	TGG TTT TAC ATT GAG TTT GG	J21
**TRAJ22rev**	AGG CCA RAC AGT CAA YTG WGT	J22, J18
**TRAJ23rev**	GGG TTT CAC AGA TAA CTC CG	J23
**TRAJ25rev**	TGG TAT GAC CAC MAC YTG GKT	J25, J7
**TRAJ26rev**	GGG CAG CAC GGA CAA TCT GG	J26
**TRAJ27rev**	TGG CTT CAC AGT GAG CGT AG	J27
**TRAJ29rev**	TGC TTT MAC ARA WAG TCT TGT	J29, J9
**TRAJ30rev**	GGG GAG AAT ATG AAG TCG TG	J30
**TRAJ31rev**	GGG CTT CAC CAC CAG CTG AG	J31
**TRAJ32rev**	TGG CTG GAC AGC AAG CAG AG	J32
**TRAJ33rev**	TGG CTT TAT AAT TAG CTT GG	J33
**TRAJ34rev**	TGG AAA GAC TTG TAA TCT GG	J34
**TRAJ37rev**	TGG TTT TAC TTG TAA AGT TG	J37
**TRAJ38rev**	CGG ATT TAC TGC CAG GCT TG	J38
**TRAJ40rev**	TGC TAA AAC CTT CAG CCT GG	J40
**TRAJ41rev**	GGG TGT GAC CAA CAG CGA GG	J41
**TRAJ42rev**	TGG TAT GAC MGA GAG TTT RGT SC	J42, J28
**TRAJ44rev**	TGG TTG CAC YTG RAG TCT TGT TC	J44, J5
**TRAJ45rev**	GGG CTG GAT GAT TAG ATG AG	J45
**TRAJ46rev**	GGG CCT AAC TGC TAA ACG AG	J46
**TRAJ47rev**	GGA CTT GAC TCT CAG AAT GG	J47
**TRAJ48rev**	TGG CCG GAT GST GAG TCT KGT YC	J48, J3
**TRAJ36rev**	GGG AAT AAY GGT GAG TCT YGT TC	J48, J36
**TRAJ49rev**	GGG TTT GAC CRT YAA MCT TGT	J49, J39
**TRAJ50rev**	AGG TTT TAC TGA TAA KCT TGT CC	J50, J14
**TRAJ52rev**	TGG ATG GAC AGT CAA GAT GG	J52
**TRAJ53rev**	TGG ATT CAC GGT TAA GAG AG	J53
**TRAJ54rev**	TGG GTG TAY AGY CAG CCT GGT YC	J54, J4
**TRAJ56rev**	TGG TCT AAC AC TCA GAG TTA	J56
**TRAJ57rev**	TGG TTT TAC TGT CAG TYT SG	J57, J43

	**BETA**	

**TRBJ1rev**	TGT GAC YGT GAG YCT GGT GC	J1-1, J2-7
**TRBJ2rev**	TGT CAC RGT KAR CCT GGT CC	J1-2, J1-6
**TRBJ3rev**	TAC AAC AGT GAG CCA ACT TC	J1-3
**TRBJ4rev**	CAG CAC WGA GAG CYG GGT YC	J1-4, J2-4
**TRBJ5rev**	TAG GAT GGA GAG TCG AGT CC	J1-5
**TRBJ2.1rev**	TAG CAC TGT SAG CCG KGT SCC TG	J2-1, J2-3
**TRBJ2.2rev**	CAG AAC CAG GAG TCC TCC GC	J2-2P
**TRBJ2.6rev**	CAG TAC GGT CAG CCT RSW GC	J2-2, J2-6
**TRBJ2.5rev**	GAG CAC CAG GAG CCG CGT GCC TG	J2-5

	**GAMMA**	

**TRGJP1rev**	AGG CGA AGT TAC TAT GAG CY	JP1,JP2
**TRGJPrev**	TGT AAT GAT AAG CTT TGT TC	JP
**TRGJ1rev**	TGT GAC AAC MAG TGT TGT TC	J1,J2

	**DELTA**	

**TRDJ1rev**	TGG TTC CAC GAT GAG TTG TG	J1
**TRDJ2rev**	TGG TTC CAC AGT CAC ACG GG	J2
**TRDJ3rev**	GGG CTC CAC GAA GAG TTT GA	J3
**TRDJ4rev**	TTG TTG TAC CTC CAG ATA GG	J4

List of optimal primer sequence as designed with the *TCRAlignment *and *TCROligo *algorithms for the TRAJ, TRBJ, TRDJ and TRGJ genes.

### RT-PCR

**Figure 1:**
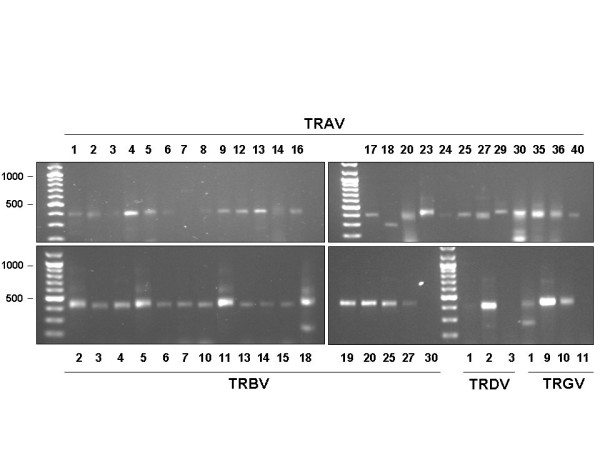
Primer validation by RT-PCR. All For primers listed in Table 1 were used together with common TR Crev primer (Table 3). Specific amplification could be seen for all primers used the only exceptions being TRAV7for, TRAV18 for and TRBV30for were positive amplification could be obtained after a second round of amplification of the first reaction.

To check whether the primers designed *in silico *were suitable to clone TR specificities, we performed RT-PCR with all the Forward primers for TRAV, TRBV, TRDV and TRGV. Each TR V primer was paired with an unique primer annealing to the 5' end of the TR C genes (Table 3). RT-PCR reactions were carried out on total RNA from peripheral blood T lymphocytes. For each reaction cDNA corresponding to approximately 1000 cells was used. As shown in figure 1 all the reactions of the TRAVfor primers produced PCR fragments of the expected size, the only exception being the TRAV7for and the TRAV18for primers. A specific TRAV7for amplification could be obtained after a second round of amplification of the first reaction. The TRAV18for primer gave a band with a lower size than expected. The TRBVfor amplifications were all positive with the expected size the only exception being the TRBV30for that could be seen after reamplification of the first reaction. Finally we got amplifications for four TRDV and TRGV for primer pairs.

**Table 3:** TR C genes reverse primers

**OLIGO NAME**	**OLIGO SEQUENCE**	**C GENE RECOGNIZED**
**TRACrev**	TCTCAGCTGGTACACGGCAG	TRAC
**TRBCrev**	AGATCTCTGCTTCTGATGGCTC	TRBC2
**TRGCrev**	GAAGGAAGAAAAATAGTGGGC	TRGC2
**TRDCrev**	GGATGGTTTGGTATGAGGCTG	TRDC

List of reverse primer sequence for TR constant regions

To confirm the specificity of the amplification products, each PCR fragment for TRVAfor and TRVBfor amplifications was purified, blunt-cloned and independently used to transform *E. coli *cells. Several random clones from each transformation were sequenced and the results are summarized in Table 4. The TR database analysis of the sequenced clones show that non-degenerate primers matching unambiguously to single TR genes selectively amplify their specific single gene targets. This specific amplification could be achieved even for very rare genes. For example the TRBV18for or TRBV11for primers selectively amplify the TRBV18 and TRBV11-1 genes that are found in 0.5% or 0.8% of circulating T cells [21], respectively.

**Table 4:** TR V primers validation

**OLIGO NAME**	**TRAV GENES OPTIMALLY RECOGNIZED**		**others found**	**N**
			
	**Experimentally found**	**Experimentally Not found**		
TRAV1for	V1-2	V1-1	-	2
TRAV2for	V2	-	-	5
TRAV3for	-	V3, V8-3	-	-
TRAV4for	V26-1	V4, V26-2	-	2
TRAV5for	V10, V34, V41	V5, V22	-	12
TRAV6for	V6	-	-	3
TRAV7for	V7	-	-	2
TRAV8for	V8-1, V8-4, V8-6	V8-2, V8-7	-	13
TRAV9for	V9-1	V9-2	-	2
TRAV12for	V12-1	V12-2, V12-3	-	2
TRAV13for	V13-1, V13-2	-	-	2
TRAV14for	V19	V14/DV4	TRDV1	4
TRAV16for	V16	V38-1, V38-2/DV8	TRB11-3	3
TRAV17for	V17	-	-	3
TRAV18for	V18	-	-	1
TRAV20for	V20, V21	-	-	5
TRAV23for	V23/DV6	-	-	5
TRAV24for	V24-	V39	V6	1
TRAV25for	V25	-	-	2
TRAV27for	V27	-	-	1
TRAV29for	V29/DV5	-	18-1	2
TRAV30for	V30	-	-	1
TRAV35for	V35	-	-	1
TRAV36for	V36/DV7	-	-	1
TRAV40for	V40	-	-	1

	**TRBV GENES OPTIMALLY RECOGNIZED**		**others found**	**N**

	**Experimentally found**	**Experimentally Not found**		

TRBV2for	V2, V23-1	V16	V6-5, V7-6, V12-5, V24-1	10
TRBV3for	V3-1, V24-1	-	-	2
TRBV4for	V4-1, V4-3, V12-5	V4-2	-	4
TRBV5for	V5-1, V5-4, V5-5, V5-6, V5-7	V5-3, V9, V5-8	-	7
TRBV6for	V6-1, V6-2, V6-5	V6-3, V6-6, V6-7, V6-8, V6-9, V17	V2	5
TRBV7for	V7-1, V7-2, V7-3, V7-4	V7-6, V7-7, V7-8, V7-9, V11-2	-	7
TRBV10for	V6-4	V10-1, V10-2, V10-3	-	1
TRBV11for	V11-1	-	-	4
TRBV13for	V12-3	V13, V12-4		7
TRBV14for	V14	V11-3	V11-2	3
TRBV15for	V15	-	-	2
TRBV18for	V18	-	-	5
TRBV19for	V19	-	-	2
TRBV20for	V29-1	V20-1	-	3
TRBV25for	V25-1	-	-	1
TRBV27for	V28	V27	-	5
TRBV30for	V30	-	-	1

Primer specificity validation by DNA sequencing. PCR products (see Fig. 1) were cloned in pTZ57R/T vector and up to thirteen clones randomly selected and sequenced. **N **= number of sequenced clones

Furthermore when analyzing clones deriving from degenerate primers, matching to a subset of TR clonotypes, we show that although sequencing a relative low number of clones, a high percentage of all possible genes were present. For example among 5 members present in the respective groups the TRAV5for or TRAV8for primers amplify 3 genes, as well the TRBV4for or TRBV5for primers amplify 3 out of 4 and 5 out of 8 genes present in the group, respectively. Interestingly, some genes amplified by degenerate primers are more frequent than other group members. This finding is likely due to the relative abundance of these transcripts within the analysed repertoires and not to amplification biases since there is no obvious relationship between primer and gene sequences.

Finally it is worth noting that some degenerate primers are also able to amplify genes that have not been computationally scored as targets (Table 1). In the case of the TRBV2for the amplified genes present only 3 to 5 base differences with the primers but were excluded in the first step of "families" generation for the presence of mismatches in the first 16 bases. The same is true for the TRBV6for primer that amplify TRBV2 gene that present only 2 nucleotides different form the primer, with one in the first 16. Although this might limit the usefulness of the primer set described for clonotypic analyses this ability increases considerably the chances to clone most TR transcripts, if not all, and turns out very useful for the creation of libraries representative of TR repertoires.

### V region Restriction enzymes analysis

The primer sets presented in this work consent the cloning of virtually the entire repertoire of TR molecules in library vectors. In the view of the creation of large TR libraries we have also analysed the frequency of restriction enzymes cutting in the database of the downloaded TR V, J and D genes. We selected 27 restriction enzymes usually used for molecular cloning and the corresponding recognition sites were used to compute a restriction map for each of our data set by employing a simple PERL program. The output is shown in Table 5 and evidences the presence of 7 enzymes (AscI, BssHII, NheI, NotI, SfiI, SacI, SalI) not cutting in any of the regions considered. These restriction enzymes could therefore be used for individual T cell or library cloning in order to avoid the loss of specific TC genes during the cloning process. Restriction sites would be added directly to the oligonucleotides based on a strategy previously described for both antibody and TC V region cloning and expression [7,22,23] that involves cloning of the engineered genes (antibody or TC V) after a leader sequence, for both bacterial (eg pelB, OmpA, phoaA) or eukaryotic (Igleader) soluble expression.

**Table 5:** Restriction enzymes cutting frequency

**Restriction enzyme name**	**Sequence cleaved**	**Number of Functional genes**										
		**TRAV**	**TRBV**	**TRDV**	**TRGV**	**TRAJ**	**TRBJ**	**TRDJ**	**TRGJ**	**TRBD**	**TRDD**	**TOTAL**
		**(47)**	**(54)**	**(3)**	**(9)**	**(50)**	**(14)**	**(4)**	**(5)**	**(2)**	**(3)**	**(191)**
ApaLI	GTGCAC	1	2	0	0	0	0	0	0	0	0	3
AscI	GGCGCGCC	**0**	**0**	**0**	**0**	**0**	**0**	**0**	**0**	**0**	**0**	**0**
BamHI	GGATCC	7	7	0	0	0	0	0	0	0	0	14
BglII	AGATCT	2	4	0	0	0	0	0	0	0	0	6
**BssHII**	**GCGCGC**	**0**	**0**	**0**	**0**	**0**	**0**	**0**	**0**	**0**	**0**	**0**
BstEII	GGTNACC	6	6	0	1	3	0	0	0	0	0	16
ClaI	ATCGAT	0	11	0	0	0	0	0	0	0	0	11
EagI	CGGCCG	0	3	0	0	0	0	0	0	0	0	3
EcoRI	GAATTC	3	0	0	1	3	0	0	0	0	0	7
EcoRV	GATATC	1	3	0	1	0	0	0	0	0	0	5
HaeIII	(AG)GCGC(CT)	4	3	0	0	1	2	0	0	0	0	10
HindIII	AAGCTT	3	4	0	0	2	1	0	1	0	0	11
KpnI	GGTACC	7	26	1	7	0	0	0	0	0	0	41
NcoI	CCATGG	3	4	0	0	0	0	0	0	0	0	7
NdeI	CATATG	6	5	0	1	0	0	0	0	0	0	12
**NheI**	**GCTAGC**	**0**	**0**	**0**	**0**	**0**	**0**	**0**	**0**	**0**	**0**	**0**
**NotI**	**GCGGCCGC**	**0**	**0**	**0**	**0**	**0**	**0**	**0**	**0**	**0**	**0**	**0**
PstI	CTGCAG	11	25	0	0	1	0	0	0	0	0	37
PvuI	CGATCG	0	1	1	0	0	0	0	0	0	0	2
SacI	GAGCTC	4	9	0	0	0	0	0	0	0	0	13
**SacII**	**CCGCGG**	**0**	**0**	**0**	**0**	**0**	**0**	**0**	**0**	**0**	**0**	**0**
**SalI**	**GTCGAC**	**0**	**0**	**0**	**0**	**0**	**0**	**0**	**0**	**0**	**0**	**0**
SmaI	CCCGGG	3	2	0	0	0	0	0	0	0	0	5
SpeI	ACTAGT	1	0	0	0	0	0	0	0	0	0	1
SphI	GCATGC	1	0	0	0	0	0	0	0	0	0	1
**SfiI**	**GGCCNNNNNGGCC**	**0**	**0**	**0**	**0**	**0**	**0**	**0**	**0**	**0**	**0**	**0**
XbaI	TCTAGA	2	5	0	0	0	0	0	0	0	0	7
XhoI	CTCGAG	0	1	0	0	0	0	0	0	0	0	1

Frequency of restriction enzymes cutting sites in human germline TR V, D and J genes. In bold the enzyme not cutting in any of the sequence analyzed.

## Discussion

The availability of databases comprising gene sequences encoding all IG or TR genes (IMGT/GENE-DB)[5] has allowed the PCR-mediated cloning of antibody repertoires or subsets of TR and has shed light over the immune response in human and mouse.

Furthermore, the engineering of synthetic antibodies has become an important methodology for the generation of reagent, diagnostic and therapeutic molecules. Obviously, the availability of databases listing all TR genes has been seen by researchers as an opportunity to do on TR what has been done with immunoglobulins. However, the cloning of TR repertoires has been hampered by a considerable higher diversity of 5' TR V genes. Several primer sets have been reported so far, but these have allowed the amplification and cloning of a restricted group of TR genes, mostly belonging to the alpha and beta chains, or have been used for the analysis of clonal T cell populations [16-19].

Here, we report a new set of primers that allow the theoretical amplification and cloning of all TR V genes. The primers were computationally designed on sequence data available at the IMGT^® ^information system, and comprising genes for all functionally synthesized TR chains. The criteria we adopted for algorithm design were such to provide the least number of primers required to amplify all catalogued genes. We obtained a number of primers considerably lower than those reported by other authors [17,19,20]. For instance, the number of primers required to amplify all V regions of TRA and TRB chains is 25 and 17, respectively, instead of 45 and 43 for each of the two amplification rounds reported by Boulter and colleagues [20].

Using two representative sets of primers matching either to single or to a subset of TR genes, we show that they can efficiently amplify target genes in one RT-PCR step, and from as little as 1000 T cells without the need of further amplifications. Among all random sequenced clones, we did not find no-TR gene sequences, a finding that confirms the selectivity of our primers. In agreement with data demonstrating the biased composition of TRA and TRB repertoires [15], we found that degenerated primers amplify with higher frequency some members of target group.

## Conclusion

Our purpose was to create a primer set able to optimally amplify all TR V genes, and we feel that we have done this. This set will allow the profiling of TR repertoire as well as the creation of libraries such as those based on single chain formats (scTR). Furthermore, the use of this set will facilitate the cloning of antigen-specific TR, a prerequisite for the development of immune-based therapies in autoimmunity, cancer and vaccination.

## Methods

### Sequences encoding TR V regions

Sequences corresponding to the functional V and J genes for TR alpha, beta, gamma and delta chains [4] were downloaded from IMGT ^® ^. 47 TRAV, 54 TRBV, 9 TRGV, 3 TRDV, 50 TRAJ, 14 TRBJ, 5 TRGJ and 4 TRDJ genes sequences were retrieved to constitute our working data set.

### Primers Design

We designed two algorithms: "*TCRAlignment*", which clusters either V or J sequences on the basis of DNA similarities; "*TCROligo*", which defines the primer set for each cluster. The parameters considered to design the algorithms were the following:

- the Forward (For) primer must anneal at the 5' end of TR V genes starting at the first base.

- the Reverse (Rev) primer must anneal at the 3'-end of TR J gene ending at the last base.

- primer length must range 19 to 23 nucleotides;

- AT content in the range of 35–65%;

- all scored primers must perfectly anneal to the last 3'-end 16 bp;

- degenerate nucleotides are introduced at no more than three positions so that the total number of different variants is less than eight, and only if it helps for full homology at the 3'-end 16 bp.

The *TCRAlignment *algorithm stores the first 23 nucleotides of each data set sequence in a N × M matrix, where N is the number of considered sequences and M is equal to 23 (maximum primer length), and generates an alignment by comparing the first reference sequence to the others. Then, the algorithm scores the alignment for sequences that differ from the first one at 1 or 2 nucleotides in the 3'end 16 bases and clusters them in a family. This criteria guarantees full homology in the 3'end region.

In order to group the large amount of similar sequences, the algorithm changes the M value by considering the four possible primer lengths (23, 22, 21, 20, 19). After counting for each length the number of homologies in the last 16 positions of each aligned sequence, the algorithm chooses, according to the previous criteria, the M value for which the number of clustered sequences is the greatest. The alignment of selected sequences is saved and the entire procedure is repeated for the remaining sequences.

For each *TCRAlignment *family, the *TCROligo *algorithm designs a primer complementary to all sequences grouped in the family. Each alignment is saved in a N × M matrix, and the algorithm designs a primer by considering each position of the alignment, that is each column of the matrix, and by filling the corresponding position of the primer as follows: for each of the first M-16 positions, where M can assume the four possible primer lengths values, the algorithm puts the nucleotide that appears most frequently in the considered column while in the last 16 positions it inserts, where necessary, degenerate nucleotides. Once the primer was designed, *TCROligo *algorithm computes its AT content and if it is not comprised between 35% and 65% the first M-16 bases of the primer are changed.

By applying this procedure to all the alignments found with the previous program we find the primers for all the functional TR V and J genes.

Common reverse primers were designed in the first exon for all the constant region and are reported in table 3

### RT-PCR

Peripheral-blood monocites cells (PBMC) were isolated from healthy donors by density gradient centrifugation (Ficoll-Paque PLUS, GE Healthcare, Milan, Italy). Total RNA was extracted from 1 × 10^6 ^cells using the E.Z.N.A. Total RNA Kit I (Omega Bio-Tek Inc.). 600 ng of RNA was reverse transcribed in a 40 μl reaction volume using the Transcriptor High Fidelity cDNA Synthesis Kit (Roche GmbH, Mannheim, Germany) and used as template for PCR (0.5–1 μl of cDNA for each reaction in 25 μl reaction volume). Common reverse primers were designed in the constant region of the alpha, beta, gamma and delta chains, and were located in the exon 1 of the respective gene. Primers were designed in order to add a BssHII restriction site on the forward and a NheI site on the reverse primer, for further cloning purposes. Amplifications conditions were 30 s at 94°C, 30 s at 52°C, and 30 s at 72°C for 35 cycles. Primers used in this study are listed in Table 1 (Biomers GmbH, Ulm, Germany). PCR products were gel-purified with the NucleoSpin Extract II kit (Macherey-Nagel GmbH, Duren, Germany) and blunt-cloned in the pTZ57R/T vector with the InsTAclone PCR cloning Kit (Fermentas Inc, Vilnius, Lithuania).

Ligations were used to transform *E. coli *DH5α cells and plated on LB/Amp/IPTG/X-gal plates for blue-white screening. For each TR group, up to 13 random clones were sequenced using a standard M13(-20) primer (5'-GTAAAACGACGGCCAGTG-3').

## Authors' contributions

DS, CS, ID conceived, designed, and coordinated the original project and provided scientific and administrative support. DC performed molecular biology procedures (PCR and cloning). IB wrote the software program and performed sequence alignments. DS and CS wrote and revised the manuscript. All authors read and approved the final manuscript.
